# Fermented Rapeseed Meal Improves Growth Performance, Antioxidant Capacity, and Intestinal Morphology of Broilers by Enhancing Nutritional Value and Reducing Antinutritional Factors

**DOI:** 10.3390/ani16030429

**Published:** 2026-01-29

**Authors:** Yinghao Liu, Shuzhen Li, Xing Chen, Xinyi Zhai, Aijuan Zheng, Zhimin Chen, Jiang Chen, Zhiheng Zou, Guohua Liu

**Affiliations:** 1 Key Laboratory for Feed Biotechnology of the Ministry of Agriculture and Rural Affairs, Institute of Feed Research, Chinese Academy of Agriculture Sciences, Beijing 100081, China; 13949080985@163.com (Y.L.);; 2 Jiangxi Province Key Laboratory of Animal Green and Healthy Breeding, Institute of Animal Husbandry and Veterinary Science, Jiangxi Academy of Agricultural Sciences, Nanchang 330200, China

**Keywords:** fermented rapeseed meal (FRSM), antinutritional factors, broiler, growth performance, antioxidant capacity

## Abstract

This study evaluated changes in nutritional components and antinutritional factors in rapeseed meal before and after microbial fermentation. It further investigated the enhancements in its nutritional value and the growth-promoting effects of fermented rapeseed meal on broiler chickens. A total of 180 one-day-old male Arbor Acres (AA) broilers were randomly allocated into 3 treatment groups, with 6 replicates per group and 10 birds per replicate. The broilers were fed a basal diet (CON), a diet with 5% soybean meal (SBM) replaced by RSM (RSM-5), or a diet with 5% SBM replaced by FRSM (FRSM-5). The date of the experiment was 28 June 2025. The results showed that FRSM improves protein quality and reduces the levels of antinutritional factors, including glucosinolates (GSL), phytic acid (PA), and condensed tannins (CT), compared with unfermented RSM. Additionally, FRSM enhances antioxidant capacity in vitro, significantly enhancing the scavenging rates of 2,2-diphenyl-1-picrylhydrazyl (DPPH) radicals, hydroxyl radicals (•OH), and superoxide anion radicals (O_2_•^−^). In the broiler feeding trial, the FRSM-5 group had significantly higher average daily gain (ADG) during the periods of 22–42 d and 1–42 d (*p* < 0.05), along with a significantly lower feed to gain ratio (F/G) (*p* < 0.05), compared with the CON and RSM-5 groups. Compared to the CON group, the FRSM-5 group showed a significantly higher slaughter rate (SR), full eviscerated rate (FER), and breast muscle rate (BMR) (*p* < 0.05), whereas the RSM-5 group had significantly lower SR and FER (*p* < 0.05). The activities of glutathione peroxidase (GSH-Px), superoxide dismutase (SOD), and total antioxidant capacity (T-AOC) in the serum and liver of the FRSM-5 group were significantly higher than those in the CON and RSM-5 groups (*p* < 0.05), and the serum immunoglobulin (IgA, IgG, and IgM) levels were significantly elevated (*p* < 0.05). Furthermore, compared with the CON and RSM-5 groups, the FRSM-5 group exhibited a significant increase in duodenal villus height (VH) (*p* < 0.05), a significant reduction in duodenal crypt depth (CD) (*p* < 0.05), and a consequent significant increase in the VH/CD (*p* < 0.05). In conclusion, microbial fermentation effectively enhances the nutritional value of RSM by improving its nutrient composition and reducing antinutritional factors. Replacing 5% SBM with FRSM in broiler diets significantly improves growth performance, enhances antioxidant capacity and immune function, and optimizes intestinal morphological structure, thereby replacing part of the soybean meal in broiler diets.

## Simple Summary

This study explored the effects of replacing part of the soybean meal in broiler diet with fermented rapeseed meal. The results showed that fermentation improved the nutritional value of rapeseed meal while reducing the content of anti-nutritional factors. Replacing part of the soybean meal in broiler diet with fermented rapeseed meal can enhance broiler growth performance, improve antioxidant capacity and immune function, and benefit intestinal morphology. Therefore, fermented rapeseed meal, as a feed ingredient, has a positive effect on the health and production of broilers. Additionally, this study provides a scientific basis for developing new protein feed resources and reducing feed costs through fermentation technology.

## 1. Introduction

Soybean meal (SBM) is the predominant protein feed ingredient in broiler diets, valued for its high crude protein (CP) content and balanced amino acid profile. However, persistent volatility in SBM prices and supply chain instability have driven the search for cost-effective alternative protein sources. Rapeseed meal (RSM), a byproduct of rapeseed oil extraction, contains approximately 40% CP and exhibits a relatively balanced amino acid composition, positioning it as a promising candidate for SBM replacement [1]. Despite its nutritional potential, RSM’s widespread application is constrained by inherent antinutritional factors (ANFs), including glucosinolates (GSL), phytic acid (PA), and condensed tannins (CT) [2]. GSL hydrolysis by myrosinase produces toxic metabolites such as isothiocyanates and thiocyanates, which interfere with thyroid iodine uptake and induce goiter [3]. PA acts as a potent chelating agent, forming indigestible complexes with essential minerals (e.g., calcium, magnesium, zinc) and amino acids, thereby reducing nutrient bioavailability [4]. CT readily binds to proteins to form insoluble aggregates, impairing protein digestion and absorption while diminishing feed palatability [5]. Numerous studies have shown that when the replacement level of RSM in broiler diets exceeds 2.5%, it can negatively affect the growth and development of broilers [6]. These limitations have hindered the large-scale utilization of RSM in broiler nutrition.

Microbial fermentation has emerged as a viable strategy to enhance RSM’s nutritional value by mitigating ANF-related constraints. Dastar et al. [7] demonstrated that using *Bacillus subtilis*, *Lactic acid bacteria*, and *Aspergillus* to ferment rapeseed meal can effectively degrade ANFs. Elbaz et al. [8] reported that FRSM exhibits an 8.21% increase in CP content, alongside 16.30% and 17.32% reductions in neutral detergent fiber and acid detergent fiber levels, respectively; GSL, PA, and CT degradation rates reached 44.51%, 37.87%, and 23.78%, respectively. Beyond nutritional optimization, FRSM has shown positive effects in broiler diets, while RSM substitution typically impairs production performance. In FRSM, ANFs are degraded, and protein quality is enhanced, which aids broilers in the digestion and absorption of nutrients. FRSM replacement improves growth outcomes and enhances the apparent digestibility of dry matter, CP, and crude fat compared to unfermented RSM [9]. Additionally, beneficial microorganisms (e.g., *Bacillus subtilis*, *Lactic acid bacteria*) and their metabolites in FRSM regulate broiler gut microbiota balance, inhibit pathogenic bacteria growth, and lower intestinal pH via organic acid production, creating a favorable environment for beneficial microbial colonization [10]. These findings establish a solid scientific foundation for FRSM application in broiler production, though further investigation into its mechanisms of action is needed to optimize the utilization of this functional feed ingredient.

In traditional FRSM preparation, *Bacillus*, *Lactic acid bacteria*, and *yeast* are usually selected for fermentation, and protease is added to improve protein quality. However, previously used fermentation strains lack specificity, and commercial protease preparations are costly. In this study, a two-stage fermentation was carried out using a crude enzyme solution of *Bacillus velezensis* with high protease production screened in our laboratory, *Lactobacillus acidophilus* that efficiently degrades GSL and PA, and *Bacillus licheniformis* that efficiently degrades CT. Since *Bacillus* produces abundant metabolic products, beneficial substances may be generated during fermentation, improving animal growth and development. Therefore, this study investigated the effects of fermented rapeseed meal on broiler growth and development.

## 2. Materials and Methods

### 2.1. Ethics Statement

This study received ethical approval from the Animal Care and Use Committee of the Institute of Feed Research of the Chinese Academy of Agricultural Science (Declaration No. IFRCAAS-20250618, Beijing, China). RSM was obtained from Yunnan Southwest Red Feed Co., Ltd. (Kunming, China). *Bacillus velezensis*, *Bacillus licheniformis*, and *Lactobacillus acidophilus* were obtained through preliminary laboratory screening.

### 2.2. Experimental Design for Fermented Rapeseed Meal

Through process optimization, the optimal fermentation process for RSM was determined. A total of 200 g of RSM was placed into a fermentation bag. Stage 1: The *Bacillus velezensis* bacterial suspension was centrifuged at 12,000 rpm and 4 °C for 20 min, and the resulting supernatant was the crude enzyme solution. The protease activity in the crude enzyme solution was 102.42 U/mL. Temperature 45 °C, pH = 7, 80 mL of *Bacillus velezensis* crude enzyme solution, moisture content 45%, enzymatic hydrolysis for 24 h. Stage 2: The solution was inoculated with 5.24 × 10^6^ CFU/g *Bacillus licheniformis* and 3.98 × 10^4^ CFU/g *Lactobacillus acidophilus*, and 50% moisture content was maintained during anaerobic fermentation in an anaerobic fermentation bag at 35 °C for 72 h. The final product obtained was FRSM. After fermentation, the sample was dried at 65 °C for 24 h to be tested.

### 2.3. Determination of Fermented Rapeseed Meal Parameters

The crude protein (GB/T 6432-2018 [11]), acid-soluble protein (NY/T 3801-2020 [12]), crude fiber (GB/T 6434-2006 [13]), and ash (GB/T 6438-2007 [14]) were determined in RSM and FRSM samples according to the national standards of the People’s Republic of China. The content of GSL [15], PA [16], and CT [17] were determined separately using the previously reported method.

### 2.4. Experimental Design and Bird Management

A total of 180 one-day-old male Arbor Acres (AA) broiler chicks were randomly placed in 18 wire cages (0.85 × 0.35 square meters) and completely randomly assigned to 3 treatment groups, with no significant difference in initial average body weight between groups. Each group consisted of 6 replicates, with 10 chicks replicated at a time. All broiler chickens were raised under consistent and optimal conditions according to the AA broiler management guidelines. The experiment was divided into two stages: the initial stage (1–21 days) and the final stage (22–42 days). The broiler chickens were placed in stacked meat cages, equipped with water dispensers and metal feeding troughs, with 16 h of light and 8 h of dark lighting per day. The chicken coop was kept at around 35 °C until the chickens reached 7 days of age. Subsequently, the temperature gradually decreased and eventually remained at 25 °C. The relative humidity remained at 60–70% from day 1 to day 14, and then at 50%. The basal diet was formulated following the agricultural industry standard NY/T 33-2004 [18], and the detailed diet composition and nutritional levels are presented in Table 1. Broilers had ad libitum access to feed and water throughout the trial. The vaccination program was implemented as follows: Marek’s disease vaccine was administered via intramuscular injection at 1 d; Newcastle disease vaccine was given via intranasal instillation at 8 d; and infectious bursal disease vaccine was delivered via drinking water at 21 d. Routine poultry house management practices were followed during the experiment. All broilers remained in good health, and no veterinary interventions were required.

### 2.5. Sample Collection

The broiler chickens were weighed on days 1, 21, and 42 of the experiment, with the number of repetitions as the unit. Based on these weight data, the average daily weight gain (ADG) was calculated. The average daily feed intake (ADFI) was determined by measuring total feed, remaining feed, and adjusting for broiler mortality rate. Subsequently, the profit-to-gain ratio (F/G) was calculated using the ADG and ADFI values.

On day 42 of the experiment, a broiler chicken was selected that was close to the average weight for each replicate, resulting in a total of 6 birds per treatment group for slaughter performance determination. Slaughter rate (SR) was defined as the percentage of carcass weight relative to live weight. Semi-eviscerated rate (SER) refers to the percentage of the weight of edible parts (retaining the heart, liver, kidneys, glandular stomach, muscular stomach [after removing contents], and abdominal fat) relative to live weight, after removing inedible parts such as the trachea, esophagus, intestines, and reproductive organs. Full eviscerated rate (FER) was calculated as the percentage of the weight of the semi-eviscerated carcass minus the heart, liver, glandular stomach, muscular stomach, fat, head, and feet relative to live weight. Breast muscle mass ratio (BMR) is the percentage of breast muscle weight relative to actual body weight, while abdominal fat mass ratio (AFR) is the ratio of abdominal fat weight to actual body weight. The determination methods of SR, SER, FER, BMR, and AFR for meat chickens follow the agricultural industry standard NY/T823-2004 [19] and previous publications in our laboratory [20].

On days 21 and 42 of the experiment, a broiler chicken was selected that was close to the average weight for each replicate, and blood samples were collected from the wing veins. The blood samples were centrifuged at 3000 rpm for 15 min, and the supernatant (serum) was collected and transferred to sterile fresh tubes, then stored at −20 °C until analysis. Serum biochemical parameters, including triglycerides (TG), total cholesterol (TC), blood urea nitrogen (BUN), glucose (GLU), aspartate aminotransferase (AST), and alanine aminotransferase (ALT), were measured using an automatic biochemical analyzer (Model 7600, Hitachi, Tokyo, Japan). Serum levels of glutathione peroxidase (GSH-Px), catalase (CAT), superoxide dismutase (SOD), malondialdehyde (MDA), total antioxidant capacity (T-AOC), immunoglobulin A (IgA), immunoglobulin G (IgG), and immunoglobulin M (IgM) were determined using enzyme-linked immunosorbent assay (ELISA) kits (purchased from Jiangsu Enzyme Immunoassay Industrial Co., Ltd., Nanjing, China).

On day 42 of the experiment, liver tissues were collected from broilers, wrapped in aluminum foil, snap-frozen in liquid nitrogen, and then transferred to a −80 °C ultra-low-temperature freezer for storage until analysis. Liver levels of GSH-Px, CAT, SOD, MDA, and T-AOC were measured using ELISA kits (purchased from Jiangsu Enzyme Immunoassay Industrial Co., Ltd.).

On the 42nd day of the experiment, one broiler chicken was randomly selected from each replicate, resulting in a total of 6 chickens in each treatment group. Under sterile conditions, the duodenum, jejunum, and ileum were excised from broiler chickens, with each intestinal segment cut into 1–2 cm in length. The intestinal contents were gently washed with phosphate-buffered saline (PBS) and then fixed in a 50 mL centrifuge tube containing 4% formaldehyde solution. Fresh 4% formaldehyde solution was replaced within 24 h after sampling to ensure effective tissue fixation. Fixed intestinal tissue undergoes pruning, dehydration, embedding, sectioning, staining, and installation. Under an optical microscope, 10 complete and well-oriented fields of view of villi and vaginal cavities were randomly selected and photographed, and analyzed using Image Pro Plus 6.0 software (Bethesda, MD, USA). The villus height (VH) and vaginal depth (CD) were measured, and the relationship between villus height and vaginal depth (VH/CD) was calculated. For specific methods, refer to the relevant published literature [21].

### 2.6. Statistical Analysis

This experiment adopted a completely randomized design. Analysis was based on the number of repetitions. A normality test, homogeneity of variance test, analysis of variance (ANOVA), and general linear model (GLM) analysis were all conducted using SAS 9.4, and Tukey’s multiple comparison test was applied. The results are presented as mean and standard error of mean (SEM). The difference is considered statistically significant, *p* < 0.05.

## 3. Results

### 3.1. Chemical Composition and Anti-Nutritional Factors

As shown in Table 2, in comparison to RSM, the CP and ASP content of FRSM increased by 10.46% and 358.02%, Crude fiber decreased by 8.56%, Crude ash decreased by 0.62%. GSL decreased by 91.54%, PA decreased by 47.14%, and CT decreased by 55.84%.

### 3.2. Free Radical Scavenging Capacity

As shown in Table 3, in comparison to RSM, the DPPH scavenging activity, the •OH scavenging activity and O_2_•^−^ scavenging activity increased by 70.63%, 41.83%, and 45.25%.

### 3.3. Growth Performance

Table 4 presents the effects of replacing SBM with RSM or FRSM on the growth performance of broilers. Compared with the CON and RSM-5 groups, the FRSM-5 group exhibited a significant increase in ADG during the periods of 22–42 d and 1–42 d (*p* < 0.05), along with a significant decrease in F/G during the same periods (*p* < 0.05). At 1–21 d, no significant differences were observed between CON, RSM-5, and FRSM-5 in terms of ADFI, ADG, and F/G (*p* > 0.05).

### 3.4. Slaughter Performance

As shown in Table 5, the effects of RSM and FRSM substitution for SBM on broiler slaughter traits were evaluated. Relative to the CON group, the FRSM-5 group had significantly higher SR, FER, and BMR (*p* < 0.05), while the RSM-5 group had significantly lower SR and FER (*p* < 0.05).

### 3.5. Serum Biochemical Indicators

Table 6 presents the effects of replacing SBM with RSM or FRSM on the serum biochemical parameters of broilers. At 21 d, the RSM-5 group had significantly elevated serum GLU, AST, and ALT concentrations (*p* < 0.05) compared with the CON and FRSM-5 groups. At 42 d, relative to the CON and FRSM-5 groups, the RSM-5 group displayed significantly reduced serum TG and TC levels (*p* < 0.05). At 42 d, serum BUN levels were significantly higher in the RSM-5 group but significantly lower in the FRSM-5 group compared with the CON group (*p* < 0.05).

### 3.6. Antioxidant Enzyme Activities and Lipid Oxidation Level

As shown in Table 7, the effects of RSM and FRSM substitution for SBM on the serum antioxidant status of broilers were evaluated at 21 and 42 d. At 21 d, relative to the CON and RSM-5 groups, the FRSM-5 group had significantly elevated serum SOD activity and T-AOC (*p* < 0.05) and a significantly reduced serum MDA level (*p* < 0.05). At 42 d, the FRSM-5 group exhibited significantly increased serum GSH-Px, SOD, and T-AOC (*p* < 0.05) and a significant decrease in serum MDA (*p* < 0.05) compared with the CON and RSM-5 groups. At 21 and 42 d, there were no significant differences in CAT levels between CON, RSM-5, and FRSM-5 (*p* > 0.05).

### 3.7. Antioxidant Status in the Liver

Table 8 presents the effects of replacing SBM with RSM or FRSM on the hepatic antioxidant capacity of broilers. At 42 d, compared with the CON and RSM-5 groups, the FRSM-5 group exhibited significantly increased hepatic GSH-Px activity, SOD activity, and T-AOC (*p* < 0.05), along with a significant decrease in MDA content in the liver (*p* < 0.05).

### 3.8. Immune Function

As shown in Table 9, the effect of RSM and FRSM on serum immunoglobulin levels was evaluated at 21 d and 42 d. At 21 d, relative to the CON and RSM-5 groups, the FRSM-5 group had significantly elevated serum IgG and IgM concentrations (*p* < 0.05). At 42 d, the FRSM-5 group exhibited significantly increased serum IgA and IgG levels (*p* < 0.05) compared with the CON and RSM-5 groups, while the RSM-5 group displayed a significant reduction in serum IgM levels compared with the CON and FRSM-5 groups (*p* < 0.05).

### 3.9. Intestinal Morphology

Table 10 presents the effects of replacing SBM with RSM or FRSM on the intestinal morphology of broilers. Compared with the CON group, the RSM-5 group exhibited a significant decrease in duodenal VH (*p* < 0.05), while the FRSM-5 group showed a significant increase in duodenal VH (*p* < 0.05). Relative to the CON and RSM-5 groups, the FRSM-5 group had a significant reduction in duodenal CD and a significant increase in duodenal VH/CD (*p* < 0.05). Additionally, compared with the CON and RSM-5 groups, the FRSM-5 group displayed a significant decrease in jejunal CD and a significant increase in jejunal VH/CD ratio (*p* < 0.05). There were no significant differences in VH, CD, and VH/CD in the ileum among CON, RSM-5, and FRSM-5 groups (*p* > 0.05).

## 4. Discussion

RSM is a promising alternative protein feed resource in poultry production [22]. However, its widespread application is limited by poor palatability and high concentrations of antinutritional factors (ANFs), including glucosinolates (GSL), phytic acid (PA), and condensed tannins (CT) [23]. Microbial fermentation has emerged as an effective strategy to address these limitations [24]. Wang et al. [25] reported that solid-state fermentation reduced RSM’s glucoside, phytic acid, crude fiber, and tannic acid contents by 99.18%, 42.41%, 27.21%, and 34.17%, respectively, while significantly increasing CP, amino acid, and peptide concentrations. Similarly, Rehemujiang et al. [26] demonstrated that synergistic fermentation of RSM with *Bacillus subtilis* and *Saccharomyces cerevisiae* enhanced DPPH radical scavenging capacity; weaned rats fed this fermented product showed elevated serum and hepatic T-SOD activity and reduced MDA levels, confirming improved systemic antioxidant capacity. *Bacillus velezensis* has strong secretion ability; the crude enzyme solution contains multiple complex enzyme systems that can break down macromolecular proteins in RSM and alter protein structures. Enzymatic treatment can efficiently release fermentable substrates, providing a better substrate foundation for subsequent microbial fermentation than traditional direct fermentation. Additionally, the screened Lactobacillus acidophilus and Bacillus licheniformis can specifically degrade GSL, PA, and CT in RSM. During fermentation, enzymatic hydrolysis and microbial fermentation are functionally complementary, and through division of labor and cooperation, they can achieve a more comprehensive nutritional improvement than single-strain fermentation. Therefore, the present study employed a two-stage processing protocol: initial enzymatic hydrolysis using crude enzyme solution from *Bacillus velezensis*, followed by mixed fermentation with *Bacillus licheniformis* and *Lactobacillus acidophilus*. Process optimization yielded FRSM with significantly increased CP and ASP contents, reduced GSL, PA, and CT levels, and enhanced scavenging capacities against DPPH radicals, •OH, and O_2_•^−^. Vlassa et al. [2] reported that using only yeast to ferment rapeseed meal can achieve a glucosinolate degradation rate of 55% and a crude protein content of 38%. In this experiment, a two-stage fermentation was used, which is significantly better than single-strain fermentation, and the content of acid-soluble protein increased, improving protein quality.

Beyond nutritional enhancement, microbial fermentation promotes nutrient absorption and utilization in animals [26]. Consistent with this, Dastar et al. [7] reported that RSM supplementation significantly reduced broiler ADG and F/G, whereas FRSM fermented with *Lactic acid bacteria*, *Bacillus subtilis*, and *Aspergillus niger* improved these growth performance indices. In the current study, replacing 5% SBM with FRSM significantly increased broiler ADG and decreased F/G, while RSM substitution showed a non-significant downward trend in ADG. This latter observation may be attributed to the moderate RSM replacement rate (5%), which is consistent with Yadav et al. [27], who noted that low RSM inclusion levels minimize adverse effects on growth. The main reason why FRSM can improve production performance is that fermentation breaks down large molecular proteins into smaller peptides and amino acids that are more easily absorbed. At the same time, the destruction of fiber structure reduces the viscosity of chyme and improves the digestibility of nutrients. This means that broiler chickens eating the same feed can absorb more effective nutrients for weight gain, thereby directly reducing the feed-to-weight ratio.

Slaughter performance is a critical indicator of broiler production efficiency, directly correlating with economic returns [28]. Wang et al. [29] reported contradictory effects of RSM substitution: while some studies show improved FER and overall slaughter performance, others indicate negative impacts. In the present study, 5% FRSM substitution improved broiler SR, FER, and BMR, whereas RSM substitution reduced SR and FER. This discrepancy underscores the importance of fermentation in mitigating RSM’s inherent drawbacks, as ANF degradation and nutrient optimization collectively promote muscle deposition and carcass quality.

Serum biochemical parameters reflect an animal’s metabolic status, tissue permeability, and organ function [30]. TG and TC are key markers of lipid metabolism [31]. Manyeula et al. [6] reported that RSM substitution gradually reduces serum TC levels with increasing inclusion rates, while Chen et al. [32] found that replacing 15% fish meal with FRSM in shrimp diets promotes TG and TC synthesis. In the current study, RSM substitution reduced serum TG and TC levels, whereas FRSM had no significant effect. This suggests that PA and CT in RSM form chelate with dietary fats, inhibiting lipid absorption [33]; fermentation degrades these ANFs, eliminating such absorption barriers. The fermentation process mainly degrades anti-nutritional factors such as GLS, PA, and CT, improving protein quality. These improvements are more directly related to nutrient utilization efficiency, while FRSM has no specific regulatory effect on lipid absorption. Therefore, there is no significant difference in TC and TG levels between the FRSM-5 and CON groups.

BUN is a key indicator of protein utilization efficiency, with lower levels reflecting enhanced protein deposition [34]. Xu et al. [35] showed that FRSM reduces broiler serum BUN levels, improving protein utilization. Consistent with this, the present study found that 5% RSM substitution significantly increased serum BUN, whereas FRSM substitution significantly decreased it. This is likely due to PA and CT in RSM binding to proteins and impairing their digestion [33]; fermentation degrades these ANFs and, via microbial proteases, breaks down macromolecular proteins into small peptides and free amino acids, enhancing digestibility and amino acid balance. As a result, absorbed amino acids are preferentially directed toward protein synthesis rather than deamination and urea production [36].

Serum glucose (GLU) is the primary energy source and a marker of carbohydrate metabolism [37]. Wu et al. [38] reported that GSL in RSM induces liver and thyroid toxicity, impairing glucose metabolism and increasing serum GLU levels in broilers. In the current study, RSM-fed broilers showed elevated serum GLU, whereas FRSM-fed birds had no significant difference compared to the control group. This is consistent with previous studies, where the GSL in RSM limits the oxidative energy supply of glucose. After microbial fermentation, GSL is degraded, relieving the restriction on glucose metabolism and restoring GLU to normal levels [33].

AST and ALT are hepatocellular enzymes; elevated serum activity indicates liver injury [39]. Ibrahim et al. [40] reported that replacing 20% SBM with RSM elevated hepatic AST and ALT activity, suggesting liver damage. In the present study, RSM-fed broilers showed significantly elevated serum AST and ALT levels, whereas FRSM-fed birds had no difference from the control. This indicates that residual GSL, PA, and CT in RSM induce hepatocellular damage, releasing these enzymes into the bloodstream; fermentation effectively degrades these harmful substances, reducing hepatocellular stress and preserving liver integrity [33].

Broilers are highly susceptible to oxidative stress, particularly in intensive farming systems [41]. The antioxidant system is critical for maintaining health and production efficiency: SOD acts as the first line of defense against reactive oxygen species (ROS), catalyzing O_2_•^−^ dismutation into hydrogen peroxide (H_2_O_2_) and oxygen [42]; GSH-Px reduces H_2_O_2_ and lipid peroxides, blocking lipid peroxidation and protecting cell membranes [43]; MDA is a lipid peroxidation byproduct and a marker of oxidative damage [44]; and T-AOC reflects the combined activity of all antioxidant substances [45]. Wu et al. [38] reported that RSM has no effect on broiler antioxidant capacity, whereas FRSM increases serum SOD and T-AOC activity and reduces MDA levels. Consistent with this, the present study found that FRSM-fed broilers had higher serum and hepatic SOD, GSH-Px, and T-AOC activity, and lower MDA levels, compared to the control and RSM groups. This is likely due to fermentation-derived antioxidants (e.g., peptides, polyphenols, flavonoids) that enhance ROS scavenging, activate antioxidant pathways, and reduce lipid peroxidation [46], as confirmed by reduced MDA levels [47].

Immunoglobulins (IgA, IgM, IgG) play key roles in immune regulation: IgA mediates mucosal defense [48], IgM is the first antibody produced in response to antigens [49], and IgG is the primary serum antibody mediating long-term immunity [50]. Wu et al. [38] reported that RSM reduces broiler immunoglobulin levels, whereas FRSM enhances immune function. In the present study, there was no significant difference in IgA at 21 days, while at 42 days, the IgA content in the FRSM-5 group was significantly increased, indicating that FRSM can regulate IgA levels, but it requires accumulation or a longer period to induce a significant increase in IgA content. The IgG levels in the FRSM-5 group increased at both 21 and 42 days, indicating that FRSM has a sustained regulatory effect on IgG. The IgM levels in the FRSM-5 group increased at 21 days, with no significant effect at 42 days, indicating that FRSM mainly stimulates early IgM production. The possible reason why FRSM has the function of regulating immunity may be due to the production of metabolites such as polysaccharides, peptides, bacteriocins, and organic acids during fermentation, which enhance immunoglobulin secretion [51].

The intestine is the primary site of nutrient digestion and absorption; VH, CD, and the VH/CD are key indicators of intestinal function [34]. VH reflects nutrient contact surface area, while CD indicates epithelial cell regeneration rate [52]; increased VH, reduced CD, and a higher VH/CD ratio are associated with improved intestinal development and absorptive capacity [53]. Qaisrani et al. [54] reported that RSM reduces broiler duodenal VH, increases CD, and lowers the VH/CD ratio, whereas Xu et al. [35] showed that FRSM enhances these indices. In the present study, RSM reduced duodenal VH, while FRSM increased duodenal VH, decreased duodenal and jejunal CD, and improved the VH/CD ratio. This is likely due to direct chemical irritation and toxicity of RSM’s GSL, PA, and CT to intestinal epithelial cells, inhibiting proliferation and differentiation and causing villus atrophy [55]; fermentation degrades these ANFs, eliminating mucosal damage and creating a favorable environment for villus growth, as evidenced by reduced CD and improved VH/CD ratio. There were no significant differences in the VH, CD, and VH/CD of the ileum among the groups. This may be because the stimulating and nutritional effects of fermentation products are mainly absorbed in the duodenum and jejunum. The jejunum is not the main site for nutrient absorption.

## 5. Conclusions

The results of this study demonstrate that microbial fermentation effectively enhances the nutritional value of RSM, reduces antinutritional factor levels, and improves its antioxidant capacity. Replacing 5% SBM with unfermented RSM in broiler diets exerts adverse effects on broiler health and performance, whereas substituting 5% SBM with FRSM significantly improves broiler growth performance, slaughter traits, enhances SOD and T-AOC activity, and reduces MDA content, increasing serum immunoglobulin levels, and improving the intestinal morphology of the duodenum and jejunum.

## Figures and Tables

**Table 1:** Composition and nutrient levels of broiler basal diets at various stages (%).

Items	1–21 Days of Age			22–42 Days of Age		
	CON	RSM-5	FRSM-5	CON	RSM-5	FRSM-5
Corn	57.11	55.97	55.97	58.19	57.04	57.04
Vegetable Oil	1.29	1.71	1.71	2.62	3.04	3.04
Soybean meal, 46% CP	31.42	27.14	27.14	26.35	22.08	22.08
Rapeseed meal	0.00	5.00	0.00	0.00	5.00	0.00
Fermented rapeseed meal	0.00	0.00	5.00	0.00	0.00	5.00
DDGS	5.00	5.00	5.00	8.00	8.00	8.00
NaCl	0.32	0.32	0.32	0.32	0.32	0.32
CaHPO_4_	1.76	1.70	1.70	1.47	1.42	1.42
Limestone	1.40	1.39	1.39	1.32	1.30	1.30
L-Lysine, 99%	0.62	0.70	0.70	0.64	0.71	0.71
DL-Methionine, 99%	0.32	0.30	0.30	0.30	0.29	0.29
L-Threonine, 99%	0.01	0.02	0.02	0.04	0.05	0.05
L-Tryptophan, 99%	0.00	0.00	0.00	0.00	0.00	0.00
Choline chloride	0.20	0.20	0.20	0.20	0.20	0.20
Premix ^1^	0.15	0.15	0.15	0.15	0.15	0.15
TiO_2_	0.40	0.40	0.40	0.40	0.40	0.40
Total	100.00	100.00	100.00	100.00	100.00	100.00
Nutrients ^2^						
Digestive energy, MJ/kg	12.55	12.55	12.55	12.76	12.76	12.76
Crude protein, %	20.00	20.00	20.00	18.00	18.00	18.00
Calcium, %	1.00	1.00	1.00	0.85	0.85	0.85
Nonphytate phosphorus	0.45	0.45	0.45	0.42	0.42	0.42
Lysine, %	1.25	1.25	1.25	1.10	1.10	1.10
Methionine, %	0.63	0.62	0.62	0.56	0.55	0.55
Threonine, %	0.80	0.80	0.80	0.72	0.72	0.72
Methionine + Cysteine, %	0.95	0.95	0.95	0.85	0.85	0.85
Tryptophan, %	0.24	0.24	0.24	0.21	0.21	0.21

^1^ The premix provided the following per kilogram diet: vitamin A 8000 IU, vitamin D3 1000 IU, vitamin E 20 IU, vitamin K_3_ 0.5 mg, vitamin B_1_ 2.0 mg, vitamin B_2_ 8 mg, vitamin B_3_ 10 mg, vitamin B_5_ 40 mg, vitamin B_6_ 3.5 mg, vitamin B_11_ 0.5 mg, vitamin B_12_ 0.01 mg, biotin 0.12 mg, Cu (as copper sulfate) 8 mg, Fe (as ferrous sulfate) 80 mg, Mn (as manganese sulfate) 60 mg, Zn (as zinc sulfate) 40 mg, Se (as sodium selenite) 0.15 mg, I (as potassium iodide) 0.7 mg. ^2^ Calculated nutrient concentrations.

**Table 2:** Comparison of chemical composition and anti-nutritional factors between RSM and FRSM.

Items ^1^	RSM ^2^	FRSM ^3^
Chemical composition		
CP, %	42.45	44.89
ASP, %	4.24	19.42
Ash, %	8.12	8.07
CF, %	24.53	22.43
Anti-nutritional factors		
GSL, μmol/g	23.41	1.98
PA, %	5.24	2.77
CT, %	2.31	1.02
Cell counts of viable bacteria		
*Bacillus*, CFU/g	9.84 × 10^7^	9.91 × 10^9^
*Lactobacillus*, CFU/g	5.21 × 10^5^	3.54 × 10^10^

^1^ CP, crude protein; ASP, acid soluble protein; Ash, Crude ash; CF, crude fiber; GSL, Glucosinolate; PA, Phytic Acid; CT, Condensed Tannin; ^2^ RSM, rapeseed meal; ^3^ FRSM, fermented rapeseed meal.

**Table 3:** Comparison of free radical scavenging capacity between RSM and FRSM.

Items ^1^ (%)	RSM ^2^	FRSM ^3^
DPPH	12.14	82.75
•OH	19.42	61.25
O_2_•^−^	23.48	68.73

^1^ DPPH, DPPH scavenging activity; •OH, Hydroxyl radical scavenging rate; O_2_•^−^, Superoxide scavenging = g activity; ^2^ RSM, rapeseed meal; ^3^ FRSM, fermented rapeseed meal.

**Table 4:** The effects of RSM and FRSM replacing SBM on the growth performance of broiler chickens.

Items ^1^	Groups ^2^			SEM ^3^	*p*-Value
	CON	RSM-5	FRSM-5		
1–21 d					
ADFI, g	59.12	59.27	59.86	0.301	0.789
ADG, g	41.20	40.73	41.32	0.301	0.362
F/G	1.44	1.46	1.45	0.026	0.331
22–42 d					
ADFI, g	154.74	153.32	155.04	1.042	0.121
ADG, g	83.06 ^b^	82.22 ^b^	86.56 ^a^	0.609	0.008
F/G	1.86 ^a^	1.87 ^a^	1.80 ^b^	0.021	0.014
1–42 d					
ADFI, g	106.51	106.11	107.14	0.703	0.412
ADG, g	61.65 ^b^	61.55 ^b^	63.87 ^a^	0.324	0.021
F/G	1.73 ^a^	1.73 ^a^	1.68 ^b^	0.017	0.017
SR, %	93.3	91.7	93.3	0.975	0.742

^1^ ADFI, average daily feed intake; ADG, average daily gain; F/C, feed to gain; SR, Survival rate. ^2^ CON, feeding basic feed group; RSM, the group fed with 5% RSM-5 instead of SBM in the basic feed; FRSM, the group fed with 5% FRSM-5 instead of SBM in the basic feed; ^a,b^ Means within a row with different superscripts are different at *p* < 0.05. ^3^ SEM, standard error of the mean (*n* = 6).

**Table 5:** The effects of RSM and FRSM replacing SBM on the slaughter performance of broiler chickens.

Items ^1^ (%)	Groups ^2^			SEM ^3^	*p*-Value
	CON	RSM-5	FRSM-5		
SR	90.63 ^b^	89.74 ^c^	91.44 ^a^	0.310	0.039
SER	83.12	82.62	83.21	0.313	0.145
FER	75.27 ^b^	74.39 ^c^	76.31 ^a^	0.330	0.041
BMR	21.83 ^b^	21.07 ^b^	22.71 ^a^	0.676	0.027
AFR	1.03	1.05	1.05	0.030	0.529

^1^ SR, Slaughter rate; SER, Semi-evisceration rate; FER, Full evisceration rate; BMR, Breast muscle rate; AFR, Abdominal fat rate. ^2^ CON, feeding basic feed group; RSM-5, the group fed with 5% RSM instead of SBM in the basic feed; FRSM-5, the group fed with 5% FRSM instead of SBM in the basic feed; ^a–c^ Means within a row with different superscripts are different at *p* < 0.05. ^3^ SEM, standard error of the mean (*n* = 6).

**Table 6:** The effects of RSM and FRSM replacing SBM on the serum biochemicals of broiler chickens.

Items ^1^	Groups ^2^			SEM ^3^	*p*-Value
	CON	RSM-5	FRSM-5		
21 d					
TG, mmol/L	7.95	7.79	7.98	0.129	0.431
TC, mmol/L	3.57	3.48	3.55	0.171	0.152
BUN, mmol/L	29.23	29.57	29.52	0.508	0.120
GLU, μmol/L	13.61 ^b^	14.62 ^a^	13.67 ^b^	0.310	0.036
AST, IU/L	318.14 ^b^	327.55 ^a^	321.19 ^b^	3.127	0.021
ALT, U/L	64.07 ^b^	67.04 ^a^	65.41 ^b^	0.903	0.019
42 d					
TG, mmol/L	5.57 ^a^	5.09 ^b^	5.59 ^a^	0.100	0.013
TC, mmol/L	6.96 ^a^	6.35 ^b^	6.93 ^a^	0.148	0.024
BUN, mmol/L	15.24 ^b^	18.43 ^a^	12.36 ^c^	0.409	0.005
GLU, μmol/L	7.74 ^b^	8.52 ^a^	7.72 ^b^	0.101	0.033
AST, IU/L	197.64	200.64	197.81	3.004	0.095
ALT, U/L	46.85	47.20	47.15	0.602	0.241

^1^ TG, triglycerides; TC, total cholesterol; BUN, blood urea nitrogen; GLU, glucose; AST, aspartate aminotransferase; ALT, alanine aminotransferase. ^2^ CON, feeding basic feed group; RSM-5, the group fed with 5% RSM instead of SBM in the basic feed; FRSM-5, the group fed with 5% FRSM instead of SBM in the basic feed; ^a–c^ Means within a row with different superscripts are different at *p* < 0.05. ^3^ SEM, standard error of the mean (*n* = 6).

**Table 7:** The effects of RSM and FRSM replacing SBM on the serum antioxidant levels of broiler chickens.

Items ^1^	Groups ^2^			SEM ^3^	*p*-Value
	CON	RSM-5	FRSM-5		
21 d					
GSH-Px, U/mL	265.93	266.38	269.33	4.761	0.289
CAT, U/mL	35.29	34.75	34.07	1.541	0.836
SOD, U/mL	96.92 ^b^	95.55 ^b^	99.77 ^a^	2.309	0.026
MDA, nmol/L	5.82 ^a^	5.89 ^a^	5.01 ^b^	0.143	0.024
T-AOC, μmol Trolox/mL	0.43 ^b^	0.44 ^b^	0.49 ^a^	0.019	0.013
42 d					
GSH-PX, U/mL	371.45 ^b^	372.03 ^b^	380.83 ^a^	4.148	0.035
CAT, U/mL	62.39	61.93	62.69	1.626	0.779
SOD, U/mL	172.93 ^b^	172.50 ^b^	179.90 ^a^	2.481	0.005
MDA, nmol/L	4.52 ^b^	4.59 ^b^	4.09 ^a^	0.122	0.022
T-AOC, μmol Trolox/mL	0.52 ^b^	0.55 ^b^	0.66 ^a^	0.039	0.008

^1^ GSH-Px, glutathione peroxidase; CAT, catalase; SOD, superoxide dismutase; MDA, malondialdehyde; T-AOC, Total Antioxidant Capacity; ^2^ CON, feeding basic feed group; RSM-5, the group fed with 5% RSM instead of SBM in the basic feed; FRSM-5, the group fed with 5% FRSM instead of SBM in the basic feed; ^a,b^ Means within a row with different superscripts are different at *p* < 0.05. ^3^ SEM, standard error of the mean (*n* = 6).

**Table 8:** The effects of RSM and FRSM replacing SBM on the liver antioxidant of broiler chickens.

Items ^1^	Groups ^2^			SEM ^3^	*p*-Value
	CON	RSM-5	FRSM-5		
GSH-PX, nmol/min/g	1664.01 ^b^	1668.34 ^b^	1695.91 ^a^	19.846	0.032
CAT, mmoL/min/g	49.75	49.76	49.89	0.654	0.525
SOD, U/g	26,428.61 ^b^	26,483.41 ^b^	27,133.38 ^a^	385.882	0.022
MDA, nmol/g	14.85 ^a^	14.87 ^a^	14.02 ^b^	0.257	0.021
T-AOC, μmol Trolox/g	5.71 ^b^	5.75 ^b^	5.92 ^a^	0.082	0.023

^1^ GSH-Px, glutathione peroxidase; CAT, catalase; SOD, superoxide dismutase; MDA, malondialdehyde; T-AOC, Total Antioxidant Capacity; ^2^ CON, feeding basic feed group; RSM-5, the group fed with 5% RSM instead of SBM in the basic feed; FRSM-5, the group fed with 5% FRSM instead of SBM in the basic feed; ^a,b^ Means within a row with different superscripts are different at *p* < 0.05. ^3^ SEM, standard error of the mean (*n* = 6).

**Table 9:** The effect of RSM and FRSM on serum immunoglobulin levels.

Items ^1^	Groups ^2^			SEM ^3^	*p*-Value
	CON	RSM-5	FRSM-5		
21 d					
IgA, μg/mL	631.50	629.27	634.63	6.515	0.797
IgG, g/mL	11.43 ^b^	11.32 ^b^	11.84 ^a^	0.244	0.047
IgM, μg/mL	1103.31 ^b^	1099.09 ^b^	1217.87 ^a^	15.159	0.009
42 d					
IgA, μg/mL	867.33 ^b^	862.28 ^b^	885.94 ^a^	13.483	0.035
IgG, g/mL	19.32 ^b^	19.31 ^b^	20.83 ^a^	0.348	0.013
IgM, μg/mL	1988.60 ^a^	1923.81 ^b^	1995.79 ^a^	19.211	0.010

^1^ IgA, Immunoglobulin A; IgG, Immunoglobulin G; IgM, Immunoglobulin M; ^2^ CON, feeding basic feed group; RSM-5, the group fed with 5% RSM instead of SBM in the basic feed; FRSM-5, the group fed with 5% FRSM instead of SBM in the basic feed; ^a,b^ Means within a row with different superscripts are different at *p* < 0.05. ^3^ SEM, standard error of the mean (*n* = 6).

**Table 10:** The effects of RSM and FRSM replacing SBM on the intestinal morphology of broiler chickens.

Items ^1^	Groups ^2^			SEM ^3^	*p*-Value
	CON	RSM-5	FRSM-5		
Duodenum					
VH, μm	1995 ^b^	1952 ^c^	2048 ^a^	29.684	0.003
CD, μm	284 ^a^	285 ^a^	275 ^b^	3.573	0.032
VH/CD	7.02 ^b^	6.85 ^b^	7.45 ^a^	0.176	0.014
Jejunum					
VH, μm	1406.33	1397.50	1404.33	10.228	0.850
CD, μm	231 ^a^	234 ^a^	218 ^b^	4.326	0.018
VH/CD	6.09 ^b^	5.97 ^b^	6.44 ^a^	0.124	0.022
Ileum					
VH, μm	1004.17	1000.33	1006.33	8.957	0.696
CD, μm	209.67	212.33	207.83	4.226	0.587
VH/CD	4.79	4.71	4.84	0.140	0.243

^1^ VH, villus height; CD, crypt depth; VH/CD, villus height/crypt depth. ^2^ CON, feeding basic feed group; RSM-5, the group fed with 5% RSM instead of SBM in the basic feed; FRSM-5, the group fed with 5% FRSM instead of SBM in the basic feed; ^a–c^ Means within a row with different superscripts are different at *p* < 0.05. ^3^ SEM, standard error of the mean (*n* = 6).

## Data Availability

The original contributions presented in this study are included in the article. Further inquiries can be directed to the corresponding author.
